# Mendelian Randomization Analysis of Systemic Iron Status and Risk of Different Types of Kidney Disease

**DOI:** 10.3390/nu16131978

**Published:** 2024-06-21

**Authors:** Jiahui Zhou, Wanting Shi, Dongya Wu, Shujie Wang, Xinhui Wang, Junxia Min, Fudi Wang

**Affiliations:** 1 The Second Affiliated Hospital, School of Public Health, Zhejiang University School of Medicine, Hangzhou 310058, China; 2 Sir Run Run Shaw Hospital, School of Public Health, Zhejiang University School of Medicine, Hangzhou 310058, China; 3 The First Affiliated Hospital, Institute of Translational Medicine, Zhejiang University School of Medicine, Hangzhou 310058, China

**Keywords:** iron, ferritin, transferrin, Mendelian randomization, immunoglobulin A nephropathy, acute kidney disease, chronic kidney disease

## Abstract

With rapid increases in incidence, diverse subtypes, and complicated etiologies, kidney disease remains a global public health problem. Iron, as an essential trace element, has pleiotropic effects on renal function and the progression of kidney diseases. A two-sample Mendelian randomization (MR) analysis was implemented to determine the potential causal effects between systemic iron status on different kidney diseases. Systemic iron status was represented by four iron-related biomarkers: serum iron, ferritin, transferrin saturation (TfSat), and total iron binding capacity (TIBC). For systemic iron status, 163,511, 246,139, 131,471, and 135,430 individuals were included in the genome-wide association study (GWAS) of serum iron, ferritin, TfSat, and TIBC, respectively. For kidney diseases, 653,143 individuals (15,658 cases and 637,485 controls), 657,076 individuals (8160 cases and 648,916 controls), and 659,320 individuals (10,404 cases and 648,916 controls) were included for immunoglobulin A nephropathy (IgAN), acute kidney disease (AKD), and chronic kidney disease (CKD), respectively. Our MR results showed that increased serum iron [odds ratio (OR): 1.10; 95% confidence interval (95% CI): 1.04, 1.16; *p* < 0.0042], ferritin (OR: 1.30; 95% CI: 1.14, 1.48; *p* < 0.0042), and TfSat (OR: 1.07; 95% CI: 1.04, 1.11; *p* < 0.0042)] and decreased TIBC (OR: 0.92; 95% CI: 0.88, 0.97; *p* < 0.0042) were associated with elevated IgAN risk. However, no significant associations were found between systemic iron status and AKD or CKD. In our MR study, the genetic evidence supports elevated systemic iron status as a causal effect on IgAN, which suggests a potential protective effect of iron chelation on IgAN patients.

## 1. Introduction

As a major global burden, kidney diseases are both a direct cause of mortality and a risk factor for other chronic diseases, especially cardiovascular disease (CVD). Nationwide population-based studies showed that more than 10% of the adult population had at least one indicator of kidney damage in the 2000s in Australia [1], Norway [2], and the US [3]. Although the primary cause of kidney disease varies in different regions, the most common causes are diabetes, hypertension, glomerulonephritis, and other and unspecified causes [4]. Clinically, kidney disease can be characterized by increases or decreases in urine output and the content of waste products in the blood (such as creatinine and urea, which are normally excreted with urine). The stages of kidney injury can be determined by eGFR and based on the existence of albuminuria [5]. When renal failure develops, hematuria and proteinuria may be the symptoms. Waste fluid in the body may be difficult to get rid of, leading to swelling, acidemia, hyperkalemia, hypocalcemia, hyperphosphatemia, and anemia, and bone health may also be affected [6,7].

Kidney diseases can be classified into different types on the basis of the Kidney Disease: Improving Global Outcomes (KDIGO) revised definition of acute kidney impairment, such as acute kidney injury (AKI) with a duration of ≤7 days [8], acute kidney disease (AKD) with a duration of ≤3 months [9], and chronic kidney disease (CKD) with a duration more than 3 months of renal impairment [10]. Immunoglobulin A nephropathy (IgAN) may present with different phenotypes and can be subdivided into IgAN with rapidly progressive glomerulonephritis (RPGN), IgAN with AKI, and IgA deposition with minimal change disease (MCD) and also has a high risk of progression to CKD. It can be seen that IgAN interacts with the three commonly defined kidney diseases [11]. As an autoimmune kidney disease, IgAN was first described by Jean Berger and his colleague Nicole Hinglais in 1968 [12]. Actually, IgAN is a pathological type of renal disease and can only be diagnosed by immunohistology with the depositions of IgA within the mesangium [13,14], and there are currently no serum or urine biomarkers that are unequivocal for the diagnosis of IgAN [11].

Many individuals with abnormal kidney function were not included within the previous criteria [15]. In order to better describe the classification of kidney diseases and enable better management of its incidence, prevalence, morbidity, and mortality, KDIGO organized a seminar in 2020 to propose the concept of AKD, which includes AKI and all kidney functions and/or structures with abnormalities that have implications for health and last ≤3 months [16]. AKD is short-term and reversible, which means a condition with recent or sudden onset. The global prevalence of AKI is about 13 million people and has approximately 1.7 million deaths annually [17]. In the United States, AKI accounts for 1–2% of all hospitalizations, and the mortality rate among ICU hospitalizations with AKI may reach 50–70% [18]. After redefinition, the patients’ incidence of AKD without AKI was three times that of AKI [15], and the incidence of acute dialysis caused by AKD is increasing, with the risk of adverse outcomes such as CKD, end-stage kidney disease (ESKD), and death on the rise [15,19,20].

Because of the criteria of AKD, once the duration of AKD illness exceeds 3 months, most patients’ diagnoses turn into CKD [21]. Unlike AKD, CKD is considered a separate, irreversible, and progressive pathological condition that often inevitably leads to ESKD [9,22,23]. The global prevalence for CKD is evaluated to be approximately 8–16%. According to the United States Renal Data System 2023 Annual Data Report (ADR) (https://usrds-adr.niddk.nih.gov/2023), 14% of adults have CKD, and more than 30% of the population aged 65 and older in the United States has some form of renal failure [24]. The prevalence of CKD was estimated at 9.1% in 2017, which was around 697.5 million cases. The number of deaths from CKD has increased to approximately 1.2 million, with a 41.5% increase in mortality across all age groups from 1990 to 2017 [5]. CKD is also considered a risk factor, risk multiplier, and mortality driver for CVD [25,26], hypertension, diabetes [4,27], and anemia [28,29]. Actually, an additional 1.4 million deaths from CVD were attributable to impaired kidney function [5].

As a kind of essential trace element, iron plays a key role in many fundamental biochemical and physiological processes. However, the reactive nature of iron also results in nonspecific interactions with other macromolecules, such as the generation of abundant free radicals via Fenton reactions, the initiation of lipid peroxidation reactions, and the activation of the iron death signaling pathway, ultimately causing damage to various tissues and organs in the body [30,31,32]. Furthermore, iron may also play a role in drug-triggered kidney injury, such as metformin-aggravated nephropathy [33]. Nevertheless, the precise causal relationship between iron and various kidney diseases remains uncertain.

The kidney is very sensitive to ischemia–reperfusion (IR), and the common ischemia–reperfusion injury (IRI) in kidneys is mostly caused by AKI and post-transplant renal allograft dysfunction [34]. At the same time, there are many experimental studies on iron and AKI in mouse models, such as iron chelator, Deferoxamine, and the ferroptosis inhibitor ferrostatin-1, which can reduce the severity of the AKI model by inhibiting the iron toxicity of hemoglobin and hemopexin in proximal tubular cells [35]. In animal models, iron plays a key pathogenic role in the occurrence and pathogenesis of AKD. A human clinical study has used various iron markers in the body to conduct correlation studies with mortality in AKI patients. They found that high concentrations of catalytic iron and low concentrations of hepcidin in the serum of AKI patients are closely related to increased mortality, suggesting that serum catalytic iron and hepcidin concentrations can be used as helpful prognostic markers in patients with AKI [36]. 

During the progression of CKD, inflammation and impaired renal clearance will increase plasma hepcidin, inhibiting duodenal iron absorption and sequestering iron in macrophages [37,38]. The altered hepcidin level can lead to a deficiency in systemic iron, a decreased iron requirement for erythropoiesis, and resistance to endogenous and exogenous erythropoietin [39,40,41]. Hepcidin-mediated iron restriction combined with impaired renal erythropoietin production leads to anemia in CKD [42]. With the aggravation of CKD, in the late stage of CKD, patients undergoing maintenance hemodialysis usually have a negative iron balance owing to reduced absorption and increased blood loss. A study has shown that, among patients undergoing hemodialysis, the use of a high-dose regimen of intravenous iron administered proactively resulted in a significantly lower risk of death or major nonfatal cardiovascular events as compared with that observed with a reactive, low-dose regimen [43]. Above all, iron is a key factor in the process of CKD. Previous Mendelian randomization (MR) data suggested a protective effect of iron on eGFR in the population, whereas a causal relationship in CKD patients has not been reported [43]. 

All different forms of kidney disease may eventually progress to severe ESKD, for which kidney replacement is the only treatment option; however, its implication is limited due to the high cost and the shortage of renal replacement services [44]. Fortunately, early detection and intervention are effective and convenient for renoprotection [45,46].

Regarding the disease outcome of kidney disease, especially for the different types of kidney disease, the effect of iron is unclear. There are no studies on the causal study of systemic iron status and IgAN or in the larger range of AKD, and although, some observational studies have realized that there is a relationship between iron and CKD, these studies did not reveal the causal relationship between iron and CKD. Therefore, we use the disease as the outcome sample and use larger disease data to conduct a more comprehensive and in-depth MR analysis of the four iron-related indicators of the different types of kidney disease outcomes. Here, the direct evidence, which our Mendelian randomization analysis provides, verifies the causality between systemic iron status and various kidney diseases.

## 2. Materials and Methods

### 2.1. Study Design

**Figure 1:**
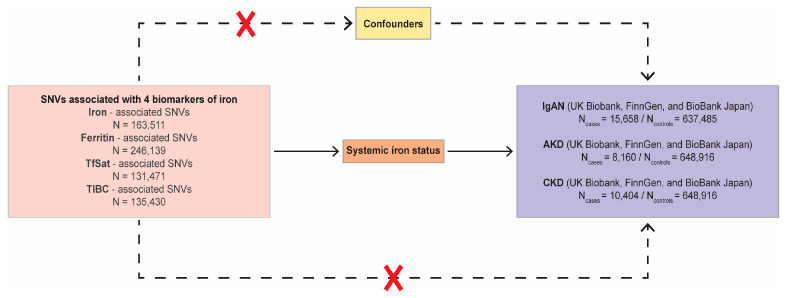
A graphic overview of the two-sample MR study design. SNVs were used as instruments of systemic iron status, and each had a genome-wide significant relation with the levels of elevated serum iron, elevated ferritin, elevated TfSat, and reduced TIBC. By utilizing genetic instruments linked to these four biomarkers of iron status, the MR method can be utilized to evaluate the causal relationship between systemic iron status and the risk of IgAN, AKD, and CKD. The red cross in the figure indicates that SNVs are unrelated to potential confounders, and not be directly associated with the outcome. AKD, acute kidney disease; CKD, chronic kidney disease; MR, Mendelian randomization; IgAN, immunoglobulin A nephropathy; SNV, single-nucleotide variation; TfSat, transferrin saturation; TIBC, total iron-binding capacity.

Our study provides a two-sample MR design to estimate the causality between systemic iron status, which was represented by four iron-related biomarkers, and three kidney diseases: IgAN, AKD, and CKD. The single-nucleotide variations (SNVs) of the four biological indicators used in the MR analysis must meet three key prerequisites: first, the instrumental variables (IVs) must be linked to exposure; second, the IVs cannot be linked to any confounding factors for the risk factor–outcome correlation; and third, the IVs cannot be directly associated with the outcome through any bypass except exposure factors. Figure 1 showed the integral research design.

### 2.2. Associations of SNVs with Systemic Iron Status

We used the summary data based on a meta-analysis of three genome-wide association studies, which were performed in the UK, Iceland, and Denmark on serum iron (n = 163,511), ferritin (n = 246,139), transferrin saturation (TfSat; n = 131,471), and total iron-binding capacity (TIBC; n = 135,430) [47]. These descriptive and statistics data for the included cohorts are exhibited in Supplementary Table S1.

Three strategies were employed for the IV selection. For strategy 1, the SNVs have to be associated with all four of the iron-related biomarkers at the same time. For strategy 2, the SNVs were related to at least one of the four biomarkers and directionally consistent for iron status with all of the other biomarkers. For strategy 3, all of the SNVs linked to any of the iron-related biomarkers were included. Because systemic iron status was considered as a whole but not individual iron biomarkers, strategy 1 was used for our main analysis. The robustness of the main analysis results was tested by the other strategies. 

### 2.3. Associations of SNVs with IgAN, AKD, and CKD

Summary statistics for IgAN, AKD, and CKD were obtained from the BioBank Japan, UK Biobank, and FinnGen databases. The study of IgAN involved 15,587 individuals from European ancestry and 71 individuals from East Asian ancestry; its control group contained 462,197 individuals of European ancestry and 175,288 individuals of East Asian ancestry. For AKD, there were 7695 cases from European ancestry and 465 cases from East Asian ancestry, and the controls of AKD included 474,571 of European ancestry and 174,345 of East Asian ancestry. In the CKD group, 8287 patients of European ancestry and 2117 patients of East Asian ancestry were involved, and 474,571 controls of European ancestry and 174,345 controls of East Asian ancestry were included [48]. The diagnosis of IgAN, AKD, and CKD was based on the 10th edition of the International Classification of Diseases (ICD-10) code. Supplementary Table S2 summarizes the characteristics of the GWAS-involved cohorts in IgAN, AKD, and CKD.

This study’s all summary statistics are publicly available. All original studies had signed informed consent and gained ethical approval of their subjects.

### 2.4. Instrument Selection

We implemented various quality control programs to identify qualified instrumental SNVs. These SNVs are from the GWAS summary data of IgAN, AKD, and CKD. First of all, in order to meet our first hypothesis, the SNVs were linked to corresponding exposures, which met with a genome-wide significance threshold of *p* = 5 × 10^−8^, and then, could be chosen as incipient IVs. For SNVs that were not available in the outcome databases, proxy SNVs were utilized, which were on the basis of the genotype data from the European population, which was adopted from the 1000 Genomes Project phase 3 (version 5) (r^2^ > 0.8).

IVs were conformed to the PLINK clustering procedure to ensure independence. The clustering technique with r^2^ < 0.01 was used to remove the SNVs linked to significant linkage disequilibrium (LD). When those SNV clusters had an r^2^ above a predetermined threshold, we only kept the SNV with the lowest *p* value. Then, the PhenoScanner database (http://www.phenoscanner.medschl.cam.ac.uk/, accessed on 5 September 2023) was utilized to find all SNVs linked to potential confounders. We eliminated all SNVs related to confounding variables and SNVs linked to outcomes to meet the second hypothesis (*p* < 5 × 10^−8^).

Due to the complex LD structure of SNVs within the human major histocompatibility complex (MHC) region, we removed SNVs within the MHC region on chromosome 6. Chromosome 6 was from the Genome Reference Conrankingium Human Build 37, hg19 [49].

Additionally, we used the following formula to calculate the *F*-statistic for each exposed SNV:

$$F=(\frac{R^{2}}{k})/(\frac{\left[1-R^{2}\right]}{\left[n-k-1\right]})$$

*R*^2^ is the exposure variation, and the explanation of *R*^2^ is the SNV. *k* is the number of SNVs. And, *n* is the sample size. The intensity of the IV was evaluated by the *F*-statistic. Generally, *F* < 10 signifies a weak instrumental bias.

Ultimately, this MR analysis’s statistical power was assessed by utilizing a Burgess design-based power calculation approach [50].

### 2.5. Mendelian Randomization Estimates

For the purpose of computing the causality of exposure variables on outcomes, genetic variation was considered as IVs in the MR analysis. In the study, by using various MR methods, we used the pooled statistics to evaluate the causalities between systemic iron status (iron, ferritin, TfSat, and TIBC) and kidney diseases. Odds ratios (ORs) for IgAN, AKD, and CKD were calculated for each standard deviation (SD) increase in the genetically predicted systemic iron status.

We utilized various univariable MR methods to evaluate the causal relationship between systemic iron status and kidney diseases, containing multiplicative random-effect inverse-variance weighted (IVW), fixed-effect IVW, weighted median, simple median, penalized weighted median, and MR-Egger approaches. Given the existence of a large number of IVs, we reckoned the random-effect IVW approach was the main analysis technique. In contrast, the robustness of the main analysis results was evaluated by the other methods of analysis.

To avoid the results being affected by the horizontal pleiotropic effects of common mutations and to make up for the shortcomings of some analytical methods that are susceptible to serious residue bias, we used another Mendelian randomization analysis using mixture model (MRMix) to evaluate the direct impact of systemic iron status on IgAN, AKD, and CKD [51].

Finally, the multivariable Mendelian randomization stem from Bayesian model averaging (MR-BMA) was employed for detection and prioritization, which were true risk factors of IgAN, AKD, and CKD from a panel of systemic iron status [52]. In this study, the approach considered all possible resultants of systemic iron status, and then, generated posterior probability (PP) for each model to compute its marginal inclusion probability (MIP) in order to determine the probability of it being a causal and decisive factor of disease risk. The model averaged causal estimate ($\hat{\theta }$_MACE_) was also computed, which can represent the average causal effect of the model containing systemic iron biomarkers. To detect useless and effective IVs, we used Cook’s distance (*Cd*) and Cochran’s *Q* statistic to quantify exception values and effective observations and to remove any SNVs with a *Q* value greater than 10 or a *Cd* greater than the related *F*-distribution median [50].

### 2.6. Sensitivity Analysis

The heterogeneity of IVs was evaluated utilizing Cochran’s *Q* statistic. Intercepts obtained from the model of MR-Egger regression were used to assess the pleiotropic effects introduced by unknown confounders [53]. A Bonferroni correction was used in multiple comparisons to minimize the false positive risk. A *p* value less than 0.0042 (0.05/12, accounting for 4 exposures and 3 outcomes) has statistical significance, while a *p* value between 0.0042 and 0.05 was considered to be of suggestive significance.

We utilized MRMix (0.1.0) packages from the statistical program R (the R Foundation for Statistical Computing; version 4.1.1) and the TwoSampleMR (version 0.5.6) to analyze the data.

## 3. Results

### 3.1. Characteristics of SNVs Used as Genetic Instruments

Increases in serum iron, ferritin, and TfSat are indicators of high iron in the body, and the high status of TIBC is the expression of a decrease in iron status in the body. According to this, we used strategy 1 to gain a total of 5, 4, and 5 independent SNVs as IVs among IgAN, AKD, and CKD, respectively, after processing through proxy selection, significance threshold screening, LD clumping, and exclusion of known pleiotropic variants (Supplementary Tables S3–S5). In strategy 1, the *F*-statistical data ranged for individual SNVs of IgAN, AKD, and CKD from 10 to 2211 (Supplementary Tables S6–S8), from 10 to 2367 in strategy 2 (Supplementary Tables S9–S11), and from 10 to 7609 in strategy 3 (Supplementary Tables S12–S14), proving that all SNVs were strong enough. Post hoc power calculations illustrated that the size of the sample involved in the present study was adequately large among the three strategies in IgAN (Supplementary Table S15), AKD (Supplementary Table S16), and CKD (Supplementary Table S17).

### 3.2. Main Analysis

**Figure 2:**
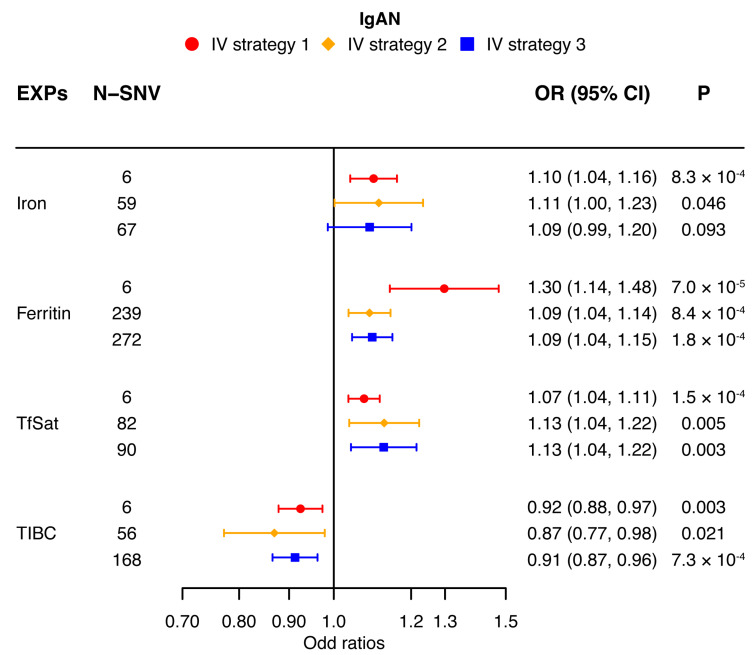
A forest plot adumbrating the MR estimates for the causal effects of iron status (multiplicative random-effect IVW) on IgAN using three strategies for IV selection. The causal effects of serum iron, ferritin, TfSat, and TIBC on IgAN risk (OR) are evaluated. The solid spots symbolize the evaluation of the causal effects, and the horizontal lines represent the 95% CIs. The strategies for the three IV sets are indicated as red circles, orange diamonds, and blue squares. 95% CI, 95% confidence interval; IVW, inverse-variance weighted; MR, Mendelian randomization; IgAN, immunoglobulin A nephropathy; OR, odds ratio; SNV, single-nucleotide variation; TfSat, transferrin saturation; TIBC, total iron-binding capacity.

**Figure 3:**
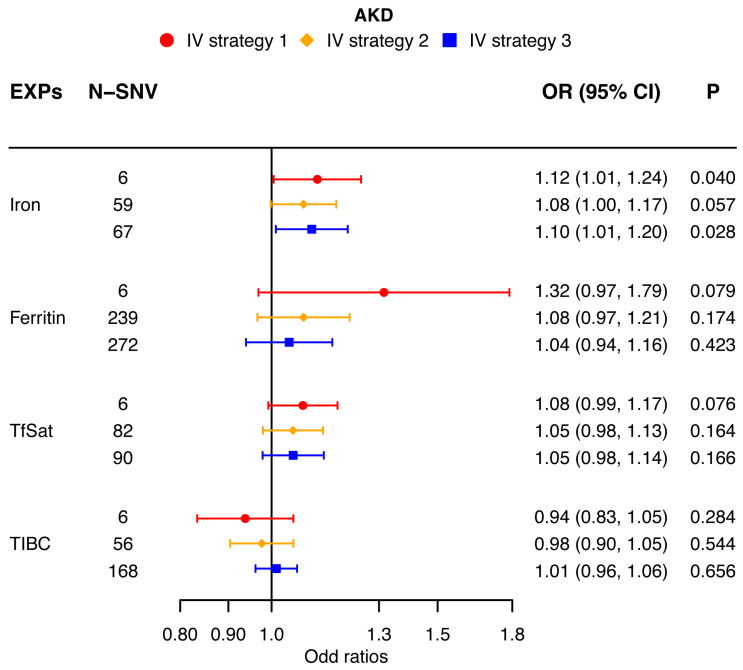
A forest plot adumbrating the MR estimates for the causal effects of iron status (multiplicative random-effect IVW) on AKD using three strategies for IV selection. The causal effects of serum iron, ferritin, TfSat, and TIBC on AKD risk (OR) were evaluated. The solid spots symbolize the evaluation of the causal effects, and the horizontal lines represent the 95% CIs. The strategies for the three IV sets are indicated as red circles, orange diamonds, and blue squares. 95% CI, 95% confidence interval; AKD, acute kidney disease; IVW, inverse-variance weighted; MR, Mendelian randomization; OR, odds ratio; SNV, single-nucleotide variation; TfSat, transferrin saturation; TIBC, total iron-binding capacity.

**Figure 4:**
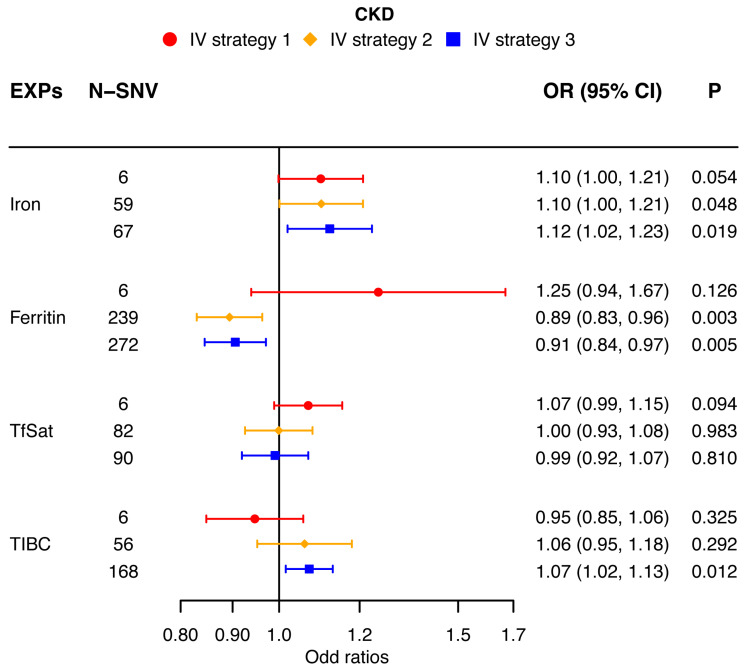
A forest plot adumbrating the MR estimates for the causal effects of iron status (multiplicative random-effect IVW) on CKD using three strategies for IV selection. The causal effects of serum iron, ferritin, TfSat, and TIBC on CKD risk (OR) were evaluated. The solid spots symbolize the evaluation of the causal effects, and the horizontal lines represent the 95% CIs. The strategies for the three IV sets are indicated as red circles, orange diamonds, and blue squares. 95% CI, 95% confidence interval; CKD, chronic kidney disease; IVW, inverse-variance weighted; MR, Mendelian randomization; OR, odds ratio; SNV, single-nucleotide variation; TfSat, transferrin saturation; TIBC, total iron-binding capacity.

Our univariable MR analysis utilizing the multiplicative random-effect IVW method derived distinct results for IgAN, AKD, and CKD. According to strategy 1, the indicators’ direction consistency and difference significance were adjusted for six independent SNVs in IgAN; the results showed significance for a high risk for IgAN [iron (OR: 1.10; 95% CI: 1.04, 1.16; *p* < 0.0042), ferritin (OR: 1.30; 95% CI: 1.14, 1.48; *p* < 0.0042), and TfSat (OR: 1.07; 95% CI: 1.04, 1.11; *p* < 0.0042)] when the systemic iron status increased, and when the systemic iron status was low, there was a risk reduction for IgAN [TIBC (OR: 0.92; 95% CI: 0.88, 0.97; *p* < 0.0042)]. The results showed that the four indicators of elevated systemic iron status have no significant causal relationship with the risk of AKD [iron (OR: 1.12; 95% CI: 1.01, 1.24; *p* > 0.0042), ferritin (OR: 1.32; 95% CI: 0.97, 1.79; *p* > 0.0042), TfSat (OR: 1.08; 95% CI: 0.99, 1.17; *p* > 0.0042), and TIBC (OR: 0.94; 95% CI: 0.83, 1.05; *p* > 0.0042)]. For CKD, the indicators’ direction consistency and difference significance were adjusted for six independent SNVs; the results showed that when the systemic iron status increased, there is no obvious enhanced risk for CKD [iron (OR: 1.10; 95% CI: 1.00, 1.21; *p* > 0.0042), ferritin (OR: 1.25; 95% CI: 0.94, 1.67; *p* > 0.0042), and TfSat (OR: 1.07; 95% CI: 0.99, 1.15; *p* > 0.0042)], and when the systemic iron status is low, the risk of CKD is not reduced significantly [TIBC (OR: 0.95; 95% CI: 0.85, 1.06; *p* > 0.0042)]. Thus, an elevated systemic iron status is a high risk for IgAN (Figure 2), and the systemic iron status has no causal relationship with AKD (Figure 3) or CKD (Figure 4).

### 3.3. Sensitivity Analysis

By utilizing the PhenoScanner database, we evaluated the potential biases as the biological pleiotropy of these instruments [54]. The phenotypes linked to the picked genetic IVs of IgAN are revealed in Supplementary Tables S18–S20, in which AKD is revealed in Supplementary Tables S21–S23 and CKD is revealed in Supplementary Tables S24–S26.

In terms of the MR-Egger regression intercept (*p* > 0.05), there was no evidence of horizontal pleiotropy among IgAN, AKD, and CKD (Supplementary Tables S27–S29). Finally, the Cochran’s *Q* statistics showed that there was no heterogeneity between the evaluated IV values of each biomarker measured utilizing the MR-Egger and IVW methods in IgAN, AKD, and CKD (Supplementary Tables S30–S32).

### 3.4. Analysis with Alternative Strategies for IV Selection

According to strategy 2 in the multiplicative random-effect IVW methods, the results show that when the systemic iron status increased, IgAN has an increased tendency [iron (OR: 1.11; 95% CI: 1.00, 1.23; *p* > 0.0042), ferritin (OR: 1.09; 95% CI: 1.04, 1.14; *p* < 0.0042), TfSat (OR: 1.13; 95% CI: 1.04, 1.22; *p* > 0.0042), and TIBC (OR: 0.87; 95% CI: 0.77, 0.98; *p* > 0.0042)] (Figure 2). The enhanced systemic iron status also has increased effects of AKD [iron (OR: 1.08; 95% CI: 1.00, 1.17; *p* > 0.0042), ferritin (OR: 1.08; 95% CI: 0.97, 1.21; *p* > 0.0042), TfSat (OR: 1.05; 95% CI: 0.98, 1.13; *p* > 0.0042), and TIBC (OR: 0.98; 95% CI: 0.90, 1.05; *p* > 0.0042)] (Figure 3) and CKD [iron (OR: 1.10; 95% CI: 1.00, 1.21; *p* > 0.0042), ferritin (OR: 0.89; 95% CI: 0.83, 0.96; *p* < 0.0042), TfSat (OR: 1.00; 95% CI: 0.93, 1.08; *p* > 0.0042), and TIBC (OR: 1.06; 95% CI: 0.95, 1.18; *p* > 0.0042)] (Figure 4), but overall, the effects are not obvious.

In strategy 3 of the multiplicative random-effect IVW approach, the results also showed that a higher systemic iron status was linked to the same elevated risk of the three kidney diseases. For IgAN, except the iron indicator of systemic iron status [iron (OR: 1.09; 95% CI: 0.99, 1.20; *p* > 0.0042)], the three indicators’ elevated effects are significant [ferritin (OR: 1.09; 95% CI: 1.04, 1.15; *p* < 0.0042), TfSat (OR: 1.13; 95% CI: 1.04, 1.22; *p* < 0.0042), and TIBC (OR: 0.91; 95% CI: 0.87, 0.96; *p* < 0.0042)] (Figure 2). But, in AKD and CKD, the results of the four indicators in systemic iron status are not significant. Elevated iron, ferritin, and TfSat have the enhanced tendencies of AKD [iron (OR: 1.10; 95% CI: 1.01, 1.20; *p* > 0.0042), ferritin (OR: 1.04; 95% CI: 0.94, 1.16; *p* > 0.0042), and TfSat (OR: 1.05; 95% CI: 0.98, 1.14; *p* > 0.0042)], and the indicator of TIBC seems to be the converse [(OR: 1.01; 95% CI: 0.96, 1.06; *p* > 0.0042)] (Figure 3). As for CKD, the indicators of ferritin, TfSat, and TIBC are the opposite of iron [iron (OR: 1.12; 95% CI: 1.02, 1.23; *p* > 0.0042), ferritin (OR: 0.91; 95% CI: 0.84, 0.97; *p* > 0.0042), TfSat (OR: 0.99; 95% CI: 0.92, 1.07; *p* > 0.0042), and TIBC (OR: 1.07; 95% CI: 1.02, 1.13; *p* > 0.0042)] (Figure 4).

Two different strategies validated the results of the main analysis, which showed that the increased systemic iron status positively associated with IgAN risk, and overall, there is no significant risk associated with AKD or CKD.

### 3.5. Analysis Using Various MR Approaches

We utilized the fixed-effect IVW, weighted median, simple median, penalized weighted median, and MR-Egger approaches as the univariable MR analysis among thte three strategies. But, for all of the above analyses, the results are consistent only in strategy 1. In the IgAN results of strategy 1, the results showed that the fixed-effect IVW, weighted median, simple median, penalized weighted median, and MR-Egger approaches could maintain a high degree of directional consistency with the results of the multiplicative random-effect IVW approach (Supplementary Tables S33–S35). However, the results of the MR-Egger analysis showed some directional inconsistency (Supplementary Tables S33–S35). For AKD and CKD of strategy 1, the results showed that the fixed-effect IVW, weighted median, simple median, penalized weighted median and MR-Egger approaches could maintain a high degree of directional consistency with the results of the multiplicative random-effect IVW approach (Supplementary Tables S33–S35).

To test and control for potential horizontal pleiotropy, the MRMix approach was employed to examine the causality. Consistently, the calculations of causal effects produced by the MRMix approach (*θ*) for serum iron, ferritin, TfSat, and TIBC were 0.065, 0.225, 0.065, and −0.085 on IgAN; 0.160, 0.470, 0.130, and 0.120 on AKD; and 0.120, 0.275, 0.080, and −0.182 on CKD, (Table 1).

Further, the non-linear MR-BMA method was implemented using IVs selected by strategy 1 to prioritize the best models for IgAN, AKD, and CKD. For IgAN, the top ranked iron biomarkers were ferritin (MIP: 0.587; $\hat{\theta }$_MACE_: 0.141), TfSat (MIP: 0.212; $\hat{\theta }$_MACE_: 0.015), iron (MIP: 0.168; $\hat{\theta }$_MACE_: 0.014), and TIBC (MIP: 0.096; $\hat{\theta }$_MACE_: −0.006), which were also involved in the best models for PP > 0.02 (Table 2). 

For AKD, the top ranked iron biomarkers were ferritin (MIP: 0.476; $\hat{\theta }$_MACE_: 0.108), iron (MIP: 0.321; $\hat{\theta }$_MACE_: 0.037), TfSat (MIP: 0.180; $\hat{\theta }$_MACE_: 0.012), and TIBC (MIP: 0.100; $\hat{\theta }$_MACE_: −0.004), which were also in the top-ranked, best individual models with PP > 0.02 (Table 3). For CKD, the top-ranked iron biomarkers were ferritin (MIP: 0.457; $\hat{\theta }$_MACE_: 0.080), iron (MIP: 0.323; $\hat{\theta }$_MACE_: 0.032), TfSat (MIP: 0.190; $\hat{\theta }$_MACE_: 0.012), and TIBC (MIP: 0.109; $\hat{\theta }$_MACE_: −0.004), which were also ranked the best individual models for PP > 0.02 (Table 4). The *Cd* and *Q*-statistic for every IV are presented in Supplementary Tables S36–S41.

## 4. Discussion

Here, we utilized MR approaches and discovered that elevated systemic iron status causally increases the risk of IgAN but is not associated with AKD or CKD risk. This finding has important implications for patient stratification with different types of kidney disease and treatment of IgAN patients in clinic. To the best of our knowledge, our study is the first to provide evidence that an elevated systemic iron status might promote the disease progression of IgAN. We used a two-sample MR study design that can generate estimates to evaluate the potential impacts of systemic iron status on IgAN, AKD, and CKD risk.

As different types of kidney diseases, the etiologies of IgAN, AKD, and CKD are different. The most common cause of AKD is malignant hypertension, followed by hematuria, nephrotoxic drug exposure, and crescents [55]. Although CKD can transition from AKD with sustained kidney injury and dysfunction for more than three months [56], there are still many pathogenic factors affecting CKD, including congenital abnormalities, exposure to toxic substances, anemia, type 2 diabetes, obesity, and metabolic acidosis [56]. A previous study has found apparent differences in the serum biomarkers between IgAN and CKD [57]; thus, as another type of kidney disease, the cause of IgAN is obviously different from that of AKD and CKD. It is mainly due to the deposition of IgA in the glomerular mesangial area, resulting in infiltration by massive immune cells [58].

The kidneys play important roles in maintaining systemic iron status. The glomeruli can reabsorb iron to prevent urinary losses of iron [59], and cells in the distal nephron of the kidney can also synthesize hepcidin to control serum iron levels [60]. The kidneys are extremely sensitive to redox balance, and iron serves as a catalyst with redox capabilities in bodily reactions [61]. More and more studies have proven that iron is related to kidney disease; perhaps, iron can provide a potential strategy for treating kidney disease. Observational studies have recognized the associations between biomarkers of iron and several autoimmune diseases, such as systemic lupus erythematosus [62], adult Still’s disease [63], and systemic juvenile rheumatoid arthritis [64], as well as IgAN [65]; however a causal relationship is yet to be defined. There is evidence from experimental and clinical studies that increased non-transferrin bound iron (NTBI) is a risk factor for the severity of AKI and poor clinical outcomes [66]. A study in a mouse model has reported that during AKI, hemopexin accumulates in the proximal tubules of the kidney, and the use of iron chelators can inhibit the harmful effects of hemopexin [35]. Interestingly, it promoted iron recycling, and erythropoiesis prevented severe-malaria-anemia-induced AKI [67]. For AKD, most studies are focus on AKI, and the results indicate that a higher level of iron is harmful for AKI. There is no clear conclusion on the causality between systemic iron status and AKD. Iron overload can cause severe oxidative stress and tissue damage, while iron deficiency can lead to anemia and other symptoms. Anemia is one of the common complications in patients who are in CKD [68], and ferumoxytol is an iron oxide nanoparticle that is approved for the treatment of CKD-associated anemia [69]. The causes of anemia in CKD patients are diverse, including EPO deficiency, iron deficiency, inflammation, and vitamin D deficiency [68,70,71]. Therefore, intravenous iron, iron supplements, and/or erythropoiesis-stimulating agents are widely used in patients with CKD [72,73,74,75,76]. Contrary to the anemia symptoms of CKD patients, tubular iron deposition is also a common feature of CKD [77]. Renin–angiotensin-converting enzyme inhibitors are also used to treat CKD patients because they may disrupt red blood cell production and inhibit dietary iron absorption [78].

Considering that most genetic variants only account for a small portion of the risk factor variation, MR studies need a relatively large sample capacity and adequate size [79]. A cross-trait meta-analysis for a disease or health condition using GWAS could identify novel genetic loci [80]. Our data included the large numbers of participants, containing the summary data from a meta-analysis of three genome-wide association studies for studying iron status [47] and the BioBank Japan, UK Biobank, and FinnGen databases for studying IgAN and CKD [48]. The public data from these databases are available concerning the relevance between genetic variants and either disease status or risk factors, therefore, providing exact estimations of genetic connections and allowing us to gain causal estimates in terms of an adequately potent MR study [81]. A previous MR study has recognized the protective effect of serum iron and ferritin on eGFR in the general population [82]. However, the causality between systemic iron status and diverse kidney diseases is still largely unknown. In our results, we provide genetic evidence for the first time for systemic iron status being a causal risk factor of IgAN, and systemic iron status was represented by the four genetically determined biomarkers serum iron, ferritin, TfSat, and TIBC. No causal association was found between systemic iron status and AKD or CKD in this study, which may due to the pleiotropic roles of iron in the development of kidney diseases. On one hand, iron accumulation can lead to kidney damage; on the other hand, anemia related to kidney disease necessitates iron supplementation for its management.

The pathogenesis of IgAN is relatively complex. Based on our main finding, systemic iron status played an important role in promoting IgAN. Although the mechanism is still unclear, previous studies could give some clues. Firstly, iron has an effect on promoting oxidative stress, inflammation, and disturbance of erythrophagocytosis. Examinations of renal biopsies have found that iron deposits, macrophage infiltration, oxidative stress markers NADPH-p22-phox- and HO-1-, and scavenger receptor CD163-positive cells were increased in IgAN patients [83]. Actually, proximal tubular epithelial cells are sensitive to the toxicity of iron, hemoglobin, and erythrophagocytosis [84]. Interestingly, a previous study detected iron deposition in 102 renal biopsy specimens from patients with various kidney diseases and found that most of the iron staining positive samples were from IgAN patients. The incidence of iron deposition in IgAN patients was 40% (12 in 30 IgAN renal biopsy specimens) [85]. Secondly, ferroptosis could also play a role in the progression of renal damage. When human mesangial cells were treated by galactose-deficient IgA1 extracted from an IgAN patient, ferroptosis was activated, accompanied by elevated ROS and malondialdehyde levels, increased structurally damaged mitochondria, and decreased GPX4 and glutathione levels. Ferrostatin-1 showed a strong rescue effect in this in vitro IgAN model [86]. Thirdly, the elevated systemic iron status could also disturb the interaction between IgA1 and the transferrin receptor (TfR) and, hence, exaggerate IgA1 mesangial deposition. TfR has been identified as an IgA1 receptor in human mesangial cells and this binding induced endocytosis [87]. The aberrant iron metabolism could alter the level of holo-transferrin (iron-saturated transferrin), sTfR (soluble forms of TfR), as well as protein glycosylation, which may affect the binding of IgA1 to mesangial TfR [88]. Indeed, co-localizations of TfR and IgA deposits in the mesangial regions of glomeruli were observed in most IgAN renal biopsies [89]. Overall, the causal effect of elevated systemic iron status on promoting IgAN could be mediated by overloaded iron, ferroptosis, and IgA1-TfR interaction. More studies on the in-depth mechanism of iron-promoted IgAN are warranted.

In recent years, numerous novel intervention methods have emerged to precisely adjust systemic iron status. In addition to iron supplementation, alternative strategies for increasing iron levels include inhibiting Hepcidin expression through epigenetic regulation [90] or using dietary flavonoid myricetin [91]. To prevent damage from iron overload, beyond iron chelation, promoting ferroportin degradation [92] or inhibiting ferroptosis [93] could also be beneficial. In this study, the findings suggest that monitoring and managing systemic iron levels in patients with kidney diseases are crucial. For patients with iron-deficient anemia associated with AKD and CKD, iron supplementation remains a safe approach. Conversely, since elevated systemic iron levels can exacerbate the development of IgAN, interventions such as iron chelation or ferroptosis inhibition may offer prognostic benefits for IgAN.

This study also has several limitations. Firstly, only summary-level statistics were used in our MR study, which made it unable to implement a stratified analysis. Secondly, the summary statistics for kidney diseases in our analysis were mainly generated from European and East Asian individuals, but not distinguishing between the two populations, which may block their translational association with a certain racial group. To assess the plausibility of the main findings, various univariable and multivariable MR approaches with different IV selection strategies were employed, yielding consistent results that further verified the robustness.

## 5. Conclusions

In this study, we examined the causal effects of systemic iron status on highly prevalent kidney diseases. Specifically, we conducted a two-sample MR study stem from summary statistics for systemic iron status and three kidney diseases, and systemic iron status was represented by four iron-related biomarkers, including IgAN, AKD, and CKD. SNVs associated with all four iron biomarkers and multiplicative random-effect IVW approaches were utilized in our main MR analysis. Other strategies for IV selection and a variety of MR approaches, including fixed-effect IVW, simple median, weighted median, MR Egger, MRMix, and MR-BMA, were also employed to evaluate the robustness for the main findings. We provide evidence that systemic iron status is a causative factor for promoting IgAN. No causality between systemic iron status and AKD or CKD was found in our analysis. Thus, this study provides new insights into iron-related intervention strategies for kidney diseases, especially IgAN.

## Figures and Tables

**Table 1:** Relationships between systemic iron status monitored by genetic instrumentation and IgAN, AKD, or CKD utilizing the six single-nucleotide variations linked to all four iron biomarkers ^1^.

Exposure ^2^	IgAN-MRMix ^3^			AKD-MRMix ^3^			CKD-MRMix ^3^		
	*θ*	π_0_	σ^2^	*θ*	π_0_	σ^2^	*θ*	π_0_	σ^2^
Iron	0.065	0.999	3.49 × 10^−4^	0.16	0.859	7.22 × 10^−4^	0.12	0.999	8.93 × 10^−4^
Ferritin	0.225	0.999	3.51 × 10^−4^	0.47	0.938	8.47 × 10^−4^	0.275	0.999	1.11 × 10^−3^
TfSat	0.065	0.999	3.49 × 10^−4^	0.13	0.973	8.75 × 10^−4^	0.08	0.999	1.05 × 10^−3^
TIBC	−0.085	0.999	3.68 × 10^−4^	0.12	0.104	2.35 × 10^−3^	−0.182	0.999	9.08 × 10^−4^

^1^ Data source and sample size: IgAN case–control of European ancestry (n = 15,587 and 462,197, respectively) study based on UK Biobank and FinnGen; IgAN case–control of East Asian ancestry (n = 71 and 175,288, respectively) study based on BioBank Japan; AKD case–control of European ancestry (n = 7695 and 474,571, respectively) study based on UK Biobank and FinnGen; AKD case–control of East Asian ancestry (n = 465 and 174,345, respectively) study based on BioBank Japan; CKD case–control of European ancestry (n = 8287 and 474,571, respectively) study based on UK Biobank and FinnGen; CKD case–control of East Asian ancestry (n = 2117 and 174,345, respectively) study stemming from BioBank Japan; genetic instruments were chosen on the basis of the three genome-wide association studies stemming from the UK, Iceland, and Denmark for iron (n = 163,511), ferritin (n = 246,139), total iron binding capacity (n = 135,430), and TfSat (n = 131,471) in blood levels. ^2^ Six SNVs for IgAN, AKD, and CKD associated with all four iron status biomarkers at genome-wide significance (*p* < 5 × 10^−8^) were employed as genetic predictors for systemic iron status. ^3^ *θ*, the estimates of causal effects generated by the MRMix approach; π_0_, the proportion of valid instrumental variables; and σ^2^, the unknown variance parameter associated with the invalid instrumental variables. Abbreviations: AKD, acute kidney disease; CKD, chronic kidney disease; IgAN, IgA nephropathy; MRMix, MR analysis utilizing a mixture model; TfSat, transferrin saturation; TIBC, total iron-binding capacity.

**Table 2:** The ranking of risk factors and models (sets of risk factors) in IgAN *.

(A) Model averaging for risk factors			
Ranking by MIP	Risk factor	MIP	$\hat{\theta }$ _MACE_
2	Ferritin	0.587	0.141
3	TfSat	0.212	0.015
1	Iron	0.168	0.014
4	TIBC	0.096	−0.006
**(B) The 10 best individual models**			
**Ranking by PP**	**Model**	**PP**	$\hat{\theta }$ **_λ_**
2	Ferritin	0.540	0.248
3	TfSat	0.180	0.071
1	Iron	0.140	0.093
4	TIBC	0.078	−0.078
2, 3	Ferritin, TfSat	0.019	0.083, 0.048
1, 2	Iron, Ferritin	0.016	0.026, 0.185
2, 4	Ferritin, TIBC	0.010	0.203, −0.017
1, 3	Iron, TfSat	0.007	−0.016, 0.082
3, 4	TfSat, TIBC	0.003	0.064, −0.010
1, 4	Iron, TIBC	0.003	0.060, −0.037

* Results were produced by utilizing the MR-BMA method. In totally, four measured systemic iron statuses were evaluated as risk factors. Four measured systemic iron statuses were genetically determined by six SNVs. The ten best individual models and the whole risk factors are shown. A positive causal estimate ($\hat{\theta }$_MACE_ or $\hat{\theta }$**_λ_**) proves a risk factor, whereas a negative value proves a protective effect, as suggested by the model. $\hat{\theta }$_MACE_ is the model averaged causal effect of a risk factor, and $\hat{\theta }$**_λ_** is the causal effect estimate for a specific model. Abbreviations: IgAN, IgA nephropathy; TfSat, transferrin saturation; TIBC, total iron-binding capacity; MIP, marginal inclusion probability; MR, Mendelian randomization; MR-BMA, MR based on Bayesian model averaging; PP, posterior probability; SNV, single-nucleotide variant.

**Table 3:** The ranking of risk factors and models (sets of risk factors) in AKD *.

(A) Model averaging for risk factors			
Ranking by MIP	Risk factor	MIP	$\hat{\theta }$ _MACE_
2	Ferritin	0.476	0.108
1	Iron	0.321	0.037
3	TfSat	0.180	0.012
4	TIBC	0.100	-0.004
**(B) The 10 best individual models**			
**Ranking by PP**	**Model**	**PP**	$\hat{\theta }$ **_λ_**
2	TIBC	0.424	0.249
1	Ferritin	0.278	0.110
3	Iron	0.146	0.076
4	TfSat	0.076	−0.064
1, 2	Iron, Ferritin	0.024	0.135, −0.070
2, 3	Ferritin, TfSat	0.015	0.011, 0.073
1, 3	Iron, TfSat	0.011	0.183, −0.056
2, 4	Ferritin, TIBC	0.011	0.362, 0.046
1, 4	Iron, TIBC	0.006	0.140, 0.035
3, 4	TfSat, TIBC	0.006	0.142, 0.090

* Results were produced by utilizing the MR-BMA method. In total, four measured systemic iron statuses were evaluated as risk factors. Four measured systemic iron statuses were genetically determined by six SNVs. The ten best individual models and the whole risk factors are shown. A positive causal estimate ($\hat{\theta }$_MACE_ or $\hat{\theta }$**_λ_**) proves a risk factor, whereas a negative value proves a protective effect, as suggested by the model. $\hat{\theta }$_MACE_ is the model averaged causal effect of a risk factor, and $\hat{\theta }$**_λ_** is the causal effect estimate for a specific model. Abbreviations: AKD, acute kidney disease; TfSat, transferrin saturation; TIBC, total iron-binding capacity; MIP, marginal inclusion probability; MR, Mendelian randomization; MR-BMA, MR based on Bayesian model averaging; PP, posterior probability; SNV, single-nucleotide variant.

**Table 4:** The ranking of risk factors and models (sets of risk factors) in CKD *.

(A) Model averaging for risk factors			
Ranking by MIP	Risk factor	MIP	$\hat{\theta }$ _MACE_
2	Ferritin	0.457	0.080
1	Iron	0.323	0.032
3	TfSat	0.190	0.012
4	TIBC	0.109	−0.004
**(B) The 10 best individual models**			
**Ranking by PP**	**Model**	**PP**	$\hat{\theta }$ **_λ_**
2	Ferritin	0.403	0.205
1	Iron	0.279	0.093
3	TfSat	0.155	0.065
4	TIBC	0.087	−0.055
1, 2	Iron, Ferritin	0.025	0.140, −0.135
2, 3	Ferritin, TfSat	0.017	−0.107, −0.095
1, 3	Iron, TfSat	0.011	0.156, −0.050
2, 4	Ferritin, TIBC	0.010	0.278, 0.030
1, 4	Iron, TIBC	0.005	0.116, 0.029
3, 4	TfSat, TIBC	0.005	0.118, 0.074

* Results were produced by utilizing the MR-BMA method. In total, four measured systemic iron statuses were evaluated as risk factors. Four measured systemic iron statuses were genetically determined by six SNVs. The ten best individual models and the whole risk factors are shown. A positive causal estimate ($\hat{\theta }$_MACE_ or $\hat{\theta }$**_λ_**) proves a risk factor, whereas a negative value proves a protective effect, as suggested by the model. $\hat{\theta }$_MACE_ is the model averaged causal effect of a risk factor, and $\hat{\theta }$**_λ_** is the causal effect estimate for a specific model. Abbreviations: CKD, chronic kidney disease; TfSat, transferrin saturation; TIBC, total iron-binding capacity; MIP, marginal inclusion probability; MR, Mendelian randomization; MR-BMA, MR based on Bayesian model averaging; PP, posterior probability; SNV, single-nucleotide variant.

## Data Availability

All data generated or analyzed during this study are included in this published article or in the data repositories listed in the References.

## Supplementary Materials

The following supporting information can be downloaded at https://www.mdpi.com/article/10.3390/nu16131978/s1, Supplementary Data: Supplementary Tables S1–S41.
